# Early dFLC response by C1D7 predicts complete hematologic response in systemic AL amyloidosis

**DOI:** 10.1007/s00277-024-06077-0

**Published:** 2024-11-12

**Authors:** Yang Liu, Jingyi Bi, Xuelin Dou, Nan Peng, Lei Wen, Yanqiu Zhao, Xiaojun Huang, Jin Lu

**Affiliations:** 1 Department of Hematology, Peking University People’s Hospital, Peking University Institute of Hematology, National Clinical Center of Hematologic Disease, Beijing, 100044 China; 2 Department of Hematology, the first affiliated Hospital of Harbin Medical University, Harbin, China; 3 Innovative Center of Hematology, Soochow University, Suzhou, Jiangsu province China

**Keywords:** AL amyloidosis, Bortezomib, Daratumumab, Rapid hematologic dFLC response

## Abstract

Daratumumab and bortezomib, the first-line drugs for AL amyloidosis, typically yield a complete hematologic response (CHR) rate of nearly 60% when used in combinations. An early achievement of CHR is crucial in amyloidosis. We retrospectively evaluated the relationship between dFLC (the difference between free light chain) reduction by Day 7 in Cycle 1 (C1D7) and CHR, organ response, and survival in 48 newly diagnosed AL amyloidosis patients receiving daratumumab, bortezomib, and dexamethasone. The CHR rate within six months was 66.7%. Using Receiver Operating Characteristic Curve curve analysis, we predicted CHR based on a dFLC reduction in C1D7 (67.0% change, optimal sensitivity 87.5%, specificity 81.3%). We introduce the novel concept of “rapid hematologic dFLC response”, defined as a reduction in dFLC levels ≥ 67% in C1D7. The CHR rate in rapid responders’ groups was higher than that in slow responders’ group (90.3% vs. 23.5%, *P*<0.01). After a median follow-up of 19 months (range: 0.3–57), the renal response rate in rapid responders was higher than that in slow responders (72.0% vs. 27.5%, *P* = 0.025). The median major organ deterioration event-free survival in the rapid responders’ group (not reached) was significantly superior to that in the slow responders’ group (19 m, 95% CI: 1.79–23.14 m, *P* = 0.048). In conclusion, early dFLC reduction in C1D7 indicates a high possibility of CHR and organ response and may allow for early modification of therapy in selected patients.

**Supplementary Information:**

The online version contains supplementary material available at 10.1007/s00277-024-06077-0.

## Key points

We propose a new concept of “the rapid dFLC response” in AL amyloidosis, based on treatment with daratumumab, bortezomib, and dexamethasone.

Rapid dFLC response in C1D7 (67.0% change, sensitivity 87.5%, specificity 81.3%) can predict complete hematologic response.

**Supplementary Information:**

The online version contains supplementary material available at 10.1007/s00277-024-06077-0.

## Introduction

Light chain amyloidosis (AL amyloidosis) is a rare plasma cell dyscrasia characterized by the deposition of insoluble amyloid fibrils in multiple organ systems [1]. The treatment of amyloidosis primarily relies on anti-plasma cell therapy and supportive care. The application of anti-plasma cell therapy has significantly improved outcomes in patients with AL amyloidosis [2]. Standard first-line therapy typically includes daratumumab, bortezomib, cyclophosphamide, and dexamethasone (Dara-BCD), achieving a complete hematologic response in nearly 60% of patients [3, 4].

The depth and speed of the hematologic response are strongly correlated with organ response and overall survival [5]. An early achievement of a complete hematologic response (CHR) is particularly crucial in cases of AL amyloidosis characterized by significant organ involvement [5–8]. The median time to a hematologic response for the daratumumab based treatment is only 7–9 days [9, 10]. We hypothesize that the hematologic response in Day 7 in Cycle 1 (referred to as C1D7) may predict the CHR rate. To explore this predictive potential, we conducted a retrospective multicentre analysis of AL amyloidosis patients treated with daratumumab and bortezomib regimens. Our study aimed to investigate the correlation between the decrease in free light chain levels in early stage of the first cycle and both the CHR and clinical outcomes.

## Materials and methods

### Patients

Medical records of 186 consecutive systemic primary AL amyloidosis patients seen at Peking University People’s Hospital and the First Affiliated Hospital of Harbin Medical University from April 2019 to November 2023 were reviewed. Among these, 80 patients received treatment with daratumumab, bortezomib, and dexamethasone, either with or without cyclophosphamide. From this group, we analysed 48 patients who had dFLC levels on C1D7 and met the criteria of having evaluable hematologic parameters, as indicated by dFLC ≥ 50 mg/L. The exclusion criteria included patients with IgM amyloidosis and those meeting the criteria for active multiple myeloma [11]. Specifically, if a patient’s plasma cell percentage in the bone marrow was greater than 10%, but without bone destruction, hypercalcemia, or free light chain ratio that exceeded 100, the patient was diagnosed with primary AL amyloidosis, consistent with a prior consensus [12]. The study was approved by the Institutional Review Board of Peking University according to the Declaration of Helsinki and all patients signed written informed consent.

### Treatment

All patients received daratumumab-based treatment. The drug was administered as an intravenous infusion at a dosage of 16 mg/kg. The treatment schedule involved weekly administrations during cycles 1 to 2. This was followed by biweekly administrations during cycles 3 to 6. Subsequently, monthly administrations were given as monotherapy. Bortezomib was administered subcutaneously. The dosage ranged from 0.7 to 1.3 mg/m^2^ on days 1, 8, 15, and 22 of each cycle, for a maximum of 6 cycles. Dexamethasone was administered intravenously at a dose of 40 mg weekly for each cycle, for a maximum of 6 cycles. For patients over 70 years of age or with severe edema, heart failure, or uncontrolled diabetes mellitus, dexamethasone could be administered at a dose of 10–20 mg per week. Some patients received cyclophosphamide orally or intravenously at a dosage of 300 mg/m^2^. All patients received prophylactic oral acyclovir to prevent herpes zoster. In our practice, we modified the treatment regimen if a patient had not achieved a partial response (PR) after one cycle, or a very good partial response (VGPR) after four cycles. This was unless there were no suitable alternative regimens available for the specific patient or if the desired organ response had been achieved. The rationale for this paradigm shift was based on a previous report. According to this report, the median time to the initial hematologic response in AL amyloidosis patients who underwent a daratumumab-based treatment was less than 1 month [10]. It was consistent with UK consensus algorithm for early treatment modification in newly diagnosed systemic light-chain amyloidosis [13]. At least 50% reduction of dFLC after the first cycle of treatment was a strong predictive factor for further VGPR/CR in AL amyloidosis patients who received bortezomib-based treatment [14]. Another rationale for this classification was based on Mayo mSMART (Mayo Stratification of Myeloma and Risk-Adapted Therapy) criteria, which stipulate that achieving at least VGPR should be reached after 4 cycles [15].

### Serum free light-chain assay

Serum free light chains were measured using the FREELITE assay kits (The Binding Site Ltd., Birmingham, UK) [16]. All patients underwent serum free light chain testing on Day 7 in Cycle 1, in addition to the routine post-treatment serum free light chain assessment.

### Response evaluation

Hematologic responses were categorized as follows: CR, VGPR, PR, NR (no response), and progressive disease (PD). The overall response rate (ORR) was calculated as the sum of the rates of CR, VGPR, and PR. Response evaluation was based on the modified International Society of Amyloidosis (ISA) criteria [17, 18]. Additionally, the graded response criteria for heart and kidney were in accordance with the literature [19]. An abnormal serum free light chain ratio caused by the suppression of the uninvolved light chain was still considered CR [3]. Additionally, for patients whose dFLC declined to less than 10 mg/L, the response was termed as a stringent dFLC response [20]. The organ response in patients with cardiac, renal, or hepatic involvement were evaluated and categorized as either improvement (organ response), stability, or progression. Organ-specific responses were assessed using the current consensus criteria. Cardiac response was evaluated with a defined baseline NT-proBNP level ≥ 650 pg/mL. Renal and hepatic response criteria were also applied. Minimal residual disease (MRD) were assessed using 8-colour flow cytometry on bone marrow aspirate after achieving CR, with the sensitivity of 1 * 10^− 4^ to 2 * 10^− 5^ based on the number of cells collected [21] .

### Follow-up

The cut-off date for follow-up was January 10th, 2024, with a median follow-up duration of 19 months. Sudden death was defined as an unexpected death in a previously clinically stable symptoms. Worsening heart failure was defined as a fatal outcome resulting from intractable heart failure, typically observed either in hospital settings or at home [22].

### Statistical analysis

For hematologic and organ responses, subjects who died before the response assessment and did not have post-baseline assessment were categorized as ‘non-responders’. The proportions of cardiac, renal, and hepatic responses were calculated by dividing the number of patients achieving each response outcome by the total number of patients with cardiac, renal and hepatic involvement, respectively. The chi-square test and Fisher’s exact test were used to compare differences between continuous variables, and the Wilcoxon signed-rank test was used for non-parametric group comparisons. The ROC curves were plotted, and the sensitivity and specificity were calculate. Survival analysis was done using the Kaplan-Meier method. Major organ deterioration progression-free survival (MOD-PFS) was defined as the time from the beginning of treatment to death, clinical manifestation of end-stage cardiac or renal failure, or hematologic progression, whichever occurs first. Major Organ Deterioration Event-Free Survival (MOD-EFS) was defined as the time from the beginning of treatment to hematologic progression, clinical manifestation of end-stage cardiac or renal disease, initiation of subsequent second-line anti–plasma cell therapy, or death, whichever occurred first. Overall survival (OS) was defined as the time from the first daratumumab treatment until death. All tests were two-tailed, and statistical significance was set at *P* < 0.05. Statistical analyses were performed with R version 4.0.2 (R Core Team, Vienna, Austria), SPSS 22.0 (SPSS Inc., Chicago, IL, USA), and GraphPad Prism 9 software (GraphPad Software Inc., La Jolla, CA, USA).

## Results

### Baseline characteristics and changes in serum free light chains

A total of 48 patients were enrolled in the study. Detailed baseline data were presented in Table 1. There were 43 patients (89.6%) with cardiac involvement, 37 patients (77.1%) with renal involvement, and 42 patients (87.5%) with ≥ 2 organs involved.

**Table 1:** Demographic and clinical characteristics

Characteristic	
Age (y)	63 (39–88)
Sex (male/female)	32/16
κ/λ light chain	14/34
Mayo 2004 stage (1/2/3a/3b)	5/12/22/9
Mayo 2012 stage (1/2/3/4)	5/10/11/22
No. of involved organs (heart/kidney/soft tissue/lung/nerve/liver/gastrointestinal)	44/37/6/5/3/4/3
dFLC at baseline (50–180/≥180 mg/L)	14/34
SPEP (g/L, ≤ 0/0–5/>5)	27/16/5
24 h-UTP (g)	1.56(0.02–17.38)
Scr (umol/L)	78.5(39–376)
ALB (g/L)	33.2(13–47)
NT-proBNP (pg/mL)	2357(33-35000)
hsTNI (pg/mL)	63.1(2-2900)
ALP (U/L)	79 (37–986)
HB (g/L)	127.5(86–155)
IVS (mm)	12 (7–25)
BMPC (%)	8 (0–40)
IgH recombination/1q21/RB1-/17p-	27/8/16/2
t(11;14)/t(4;14)/t(14;16)/others IgH recombination	20/4/2/2
1q21 triploid/> triploid	5/3

Note: All continuous variables were represented by median and range. dFLC: difference between free light chain; SPEP, Serum protein electrophoresis; 24 h-UTP: 24 h urine total protein; Scr: Serum Creatinine; ALB: albumin; NT-proBNP: N-terminal B-type natriuretic peptide precursor; hsTNI: High-sensitive troponin I; ALP: alkaline phosphates; HB: hemoglobulin; IVS: interventricular septal thickness; BMPC: bone marrow plasma cell; IgH: immunoglobulin heavy chain

**Fig. 1:**
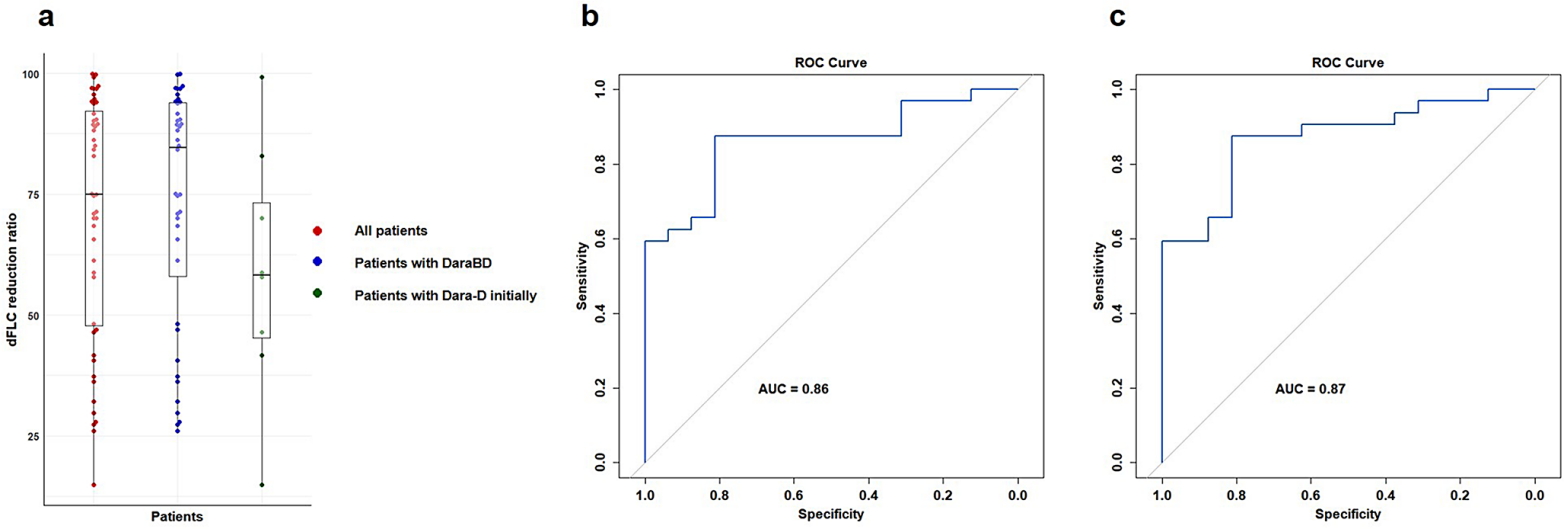
DFLC Changes in C1D7 and ROC curve analysis for the prediction of complete hematologic response by dFLC change in C1D7. (**a**) dFLC changes in C1D7 for all patients (red), patients who received the first dose of daratumumab in combination with bortezomib and dexamethasone (blue), patients who only received daratumumab, dexamethasone but did not receive bortezomib initially (green). (**b**) ROC curve to determine the optimal cutoff for predicting complete hematologic response. The AUC was 0.86, and a cutoff value of 67.0% was found to be the best for distinguishing between CHR and non-CHR patients. The sensitivity and specificity were 87.5% and 81.3%, respectively. (**c**) ROC curve to determine the optimal cutoff for predicting stringent hematologic dFLC response. dFLC, difference between free light chain; ROC, receiver operating characteristic; AUC, area under the curve

The median treatment cycles were 6 (range, 1–24). 40 patients (83.3%) received daratumumab in combination with bortezomib and dexamethasone from the outset, while 8 patients (16.7%) initially received daratumumab and dexamethasone later supplemented with bortezomib during the course of treatment. Additionally, 16 patients were treated with a regimen containing cyclophosphamide. At baseline, the median dFLC level was 367.25 mg/L (range, 60.87–4356), with 34 out of 48 patients exhibiting dFLC levels ≥ 180 mg/L. The median dFLC change by C1D7 after the initiation of daratumumab was 75.0%. For patients who received the first dose of daratumumab in combination with bortezomib, the median change in dFLC change in C1D7 was 84.6%, compared to 58.3% for those who only received daratumumab monotherapy initially. The precise changes in dFLC before and after the first dose of daratumumab was illustrated in Fig. 1a.

### Hematologic and organ response

**Fig. 2:**
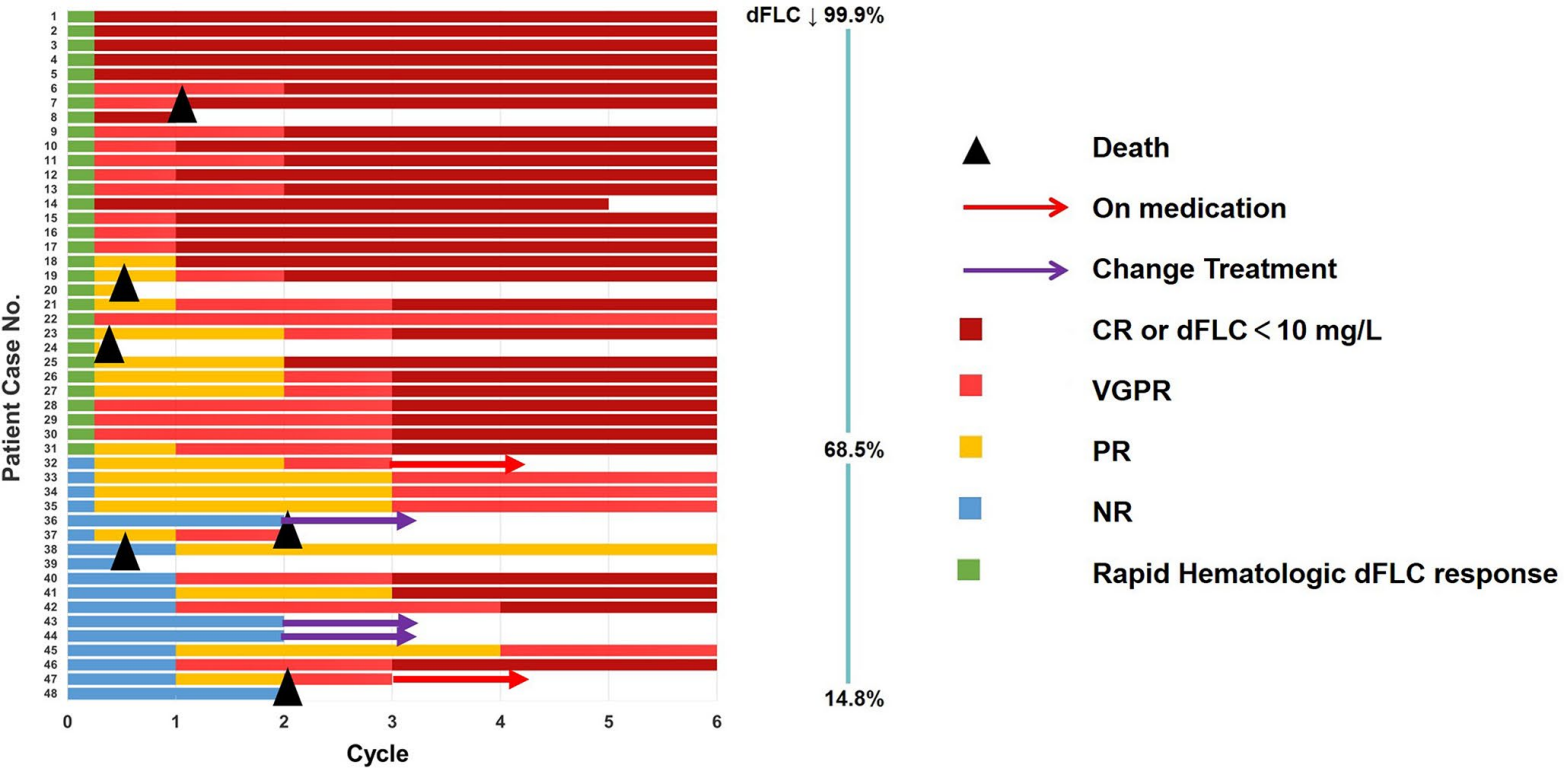
Hematologic response in Rapid-Respond Group vs. Slow-Respond Group. Swim lane plot was distributed from highest (99.9%) to lowest (14.8%) in proportion to the decrease in dFLC in C1D7. Green represented rapid hematologic dFLC response (>67%) and blue represented slow hematologic dFLC response (<67%). CR, complete response; VGPR, very good partial response; PR, partial response; NR, no response; dFLC, difference between free light chain

The CHR rate for the entire cohort was 66.7% (32/48) within six months. Similarly, the stringent dFLC response rate which defined as dFLC < 10 mg/L was also 66.7%. The swim lane was presented in Fig. 2. Furthermore, the median time to initial overall hematologic response among these patients was C1D7 (range, C1D7-C1D31), while the median time to achieve the best hematologic response was 2 months (range, 0.2-9).

Among patients with a baseline 50 ≤ dFLC<180 mg/L, 10 out of 14 patients (71.4%) achieved CHR, while 11 patients (78.6%) achieved stringent dFLC response within 6 months. Among patients with baseline dFLC ≥ 180 mg/L, 22 out of 34 patients (64.7%) achieved CHR while 21 patients (61.8%) attained stringent dFLC response within the same duration.

Among patients who received the first dose of daratumumab in combination with bortezomib and dexamethasone, 29 out of 40 patients (72.5%) achieved CHR, while 28 patients (70%) achieved stringent dFLC response within a six-month period. In contrast, among patients who did not receive bortezomib initially, 3 out of 8 patients (37.5%) achieved CHR, with 4 patients (50%) achieving stringent dFLC response within the same duration.

Out of the 41 evaluable patients with cardiac involvement, 25 (60.9%) exhibited a cardiac response at 6 months. Similarly, among the 36 evaluable patients with renal involvement, 21 (58.3%) achieved a renal response at 6 months. None of the four patients with hepatic involvement achieved an organ response.

### ROC curves for predicting efficacy based on free light chain results

In our study, a ROC curve analysis was performed to determine the optimal threshold for the dFLC reduction in C1D7 post-treatment, which best predicted CHR within six cycles of treatment.

As shown in Fig. 1b, the area under the curve (AUC) of the change in dFLC at D7 post-treatment was 0.86, indicating high predictive accuracy for CHR. The optimal sensitivity was 87.5%, while the specificity was 81.3%, when the change in dFLC was set as 67.0%. As shown in Fig. 1c, at the same threshold 67.0%, the AUC for predicting stringent dFLC response after six treatment cycles was 0.87, with a corresponding sensitivity of 87.5% and specificity of 81.3%.

### Comparisons of baseline characteristics between the two groups

Based on the threshold derived from the ROC curve analysis, patients were categorized into two groups: Rapid responders and Slow responders. Table 2 showed that there were 31 patients in the rapid responder group and 17 patients in the slow responder group. No significant differences in baseline characteristics were observed between these two groups.

**Table 2:** Clinical characteristics in rapid respond group and slow respond group

	Rapid Responders (*n* = 31)	Slow Responders (*n* = 17)	*P*
Age (y) (median, range)	61(39–75)	64(46–88)	0.257
Male	22/31	10/17	0.524
λ light chain	25/31	9/17	0.055
Mayo 2004 stage (1/2/3a/3b)	5/8/13/5	0/4/9/4	0.337
Mayo 2012 stage (1/2/3/4)	4/7/6/14	1/3/5/8	0.767
≥ 2 organ	83.9% (26/31)	88.2% (15/17)	0.682
dFLC at baseline (mg/L)	367.25 (60.87–4356)	317.5 (78.24-1541.3)	0.872
dFLC ≥ 180 mg/L	74.2% (23/31)	64.7% (11/17)	0.489
24hUTP (g)	2.67 (0.02–17.38)	1.19 (0.09–6.75)	0.051
Scr (umol/L)	76 (39–241)	81 (53–376)	0.574
ALB (g/L)	32.4 (13–47)	35.15 (19.4–43.7)	0.225
NT-proBNP (pg/mL)	2078 (33->35000)	4800 (203.7->35000)	0.804
hsTNI (pg/mL)	62.2 (2-2900)	57.8 (4-1228)	0.518
ALP (U/L)	78 (37–463)	77.5 (44–986)	0.369
HB (g/L)	127 (105–155)	129 (86–151)	0.291
IVS	12.5 (7–21)	12 (9–25)	0.879
Bone marrow PCs	7.5 (0-33.5)	8 (0–40)	0.553
t(11;14)	41.9% (13/31)	41.9% (7/17)	0.959
1q21 +	12.9% (4/31)	23.5% (4/17)	0.428

Note: All continuous variables were represented by median and range. dFLC: difference between free light chain; 24hUTP: 24 h urine total protein; Scr: Serum Creatinine; ALB: albumin. NT-proBNP: N-terminal B-type natriuretic peptide precursor; hsTNI: high sensitive troponin I; ALP: alkaline phosphates; HB: hemoglobulin; IVS: interventricular septal thickness; PC: plasma cell;

### Comparison of response and survival between rapid-response group and slow-respond group

The hematologic and organ efficacy among rapid responders was significantly higher compared to slow responders. Statistically significant differences were observed between the two groups in CHR rates, rates of dFLC<10 mg/L, and renal response. The efficacy outcomes were detailed in Table 3. The grading of cardiac and renal response between the rapid response and slow response groups is listed in supplementary Table 1.

**Table 3:** Hematologic and organ response in Rapid Respond group and Slow Respond group

	Rapid Responders (*n*/%)	Slow Responders (*n*/%)	*P*
CHR	28(90.3)	4(23.5)	<0.01
Stringent dFLC response	28(90.3)	4(23.5)	<0.01
MRD negativity	9/14	4/4	0.234
Cardiac response	18(72.0)	7(43.8)	0.104
Renal response	18(72.0)	3(27.5)	0.025
Hepatic response	0	0	NS

CHR, complete hematologic response; dFLC, difference between free light chain; MRD, minimal residual disease; NS, not significance

The median follow-up time was 19 months (range, 0.3–57 m). During the follow-up period, seven patients (14.6%) died. Among these deaths, three were sudden deaths, three were classified as worsening heart failure, and one patient died of gastrointestinal bleeding. Additionally, one surviving patient was diagnosed with end-stage cardiac failure. None of the responding patients relapsed nor progressed before completion of the planned 6-month treatment.

**Fig. 3:**
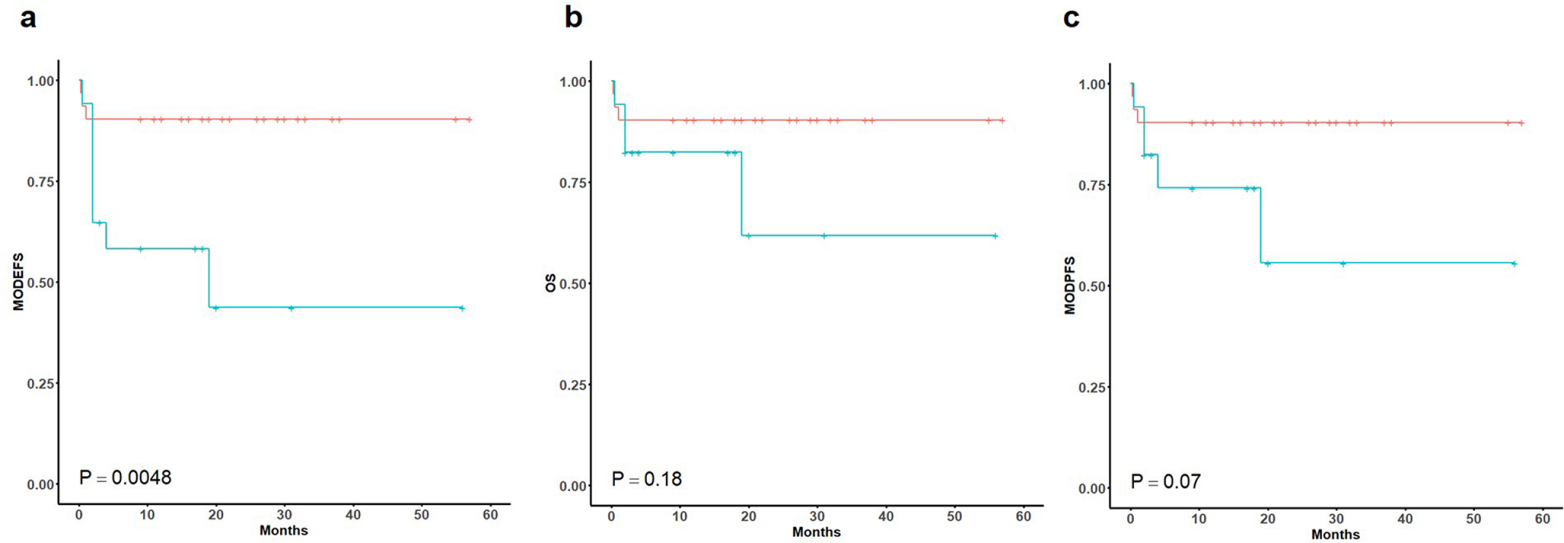
Survival curves in Rapid-Respond Group vs. Slow-Respond Group. Survival curve in patients with rapid hematologic dFLC response (orange line) and slow response group (blue-green line). **a**, MOD-EFS; **b**. MOD-PFS; **c**. OS. MOD-EFS, major organ deterioration event-free survival; MOD-PFS, major organ progression-free survival; OS, overall survival; dFLC, difference between free light chain

Figure 3a showed that the median MOD-EFS of rapid hematologic dFLC response patients (not reached) was significantly better than that of slow responders (19 m, 95% CI, 1.785–23.14 m) (*P* = 0.048). The median OS (Fig. 3b) and MOD-PFS (Fig. 3c) of rapid-dFLC response and slow hematologic dFLC response groups were not reached. The estimated 2-year OS rates were 90.3% (95% CI, 80.4–100%) for rapid hematologic dFLC response patients and 61.8% (95% CI, 33.7–100%) for slow hematologic dFLC response patients (*P* = 0.18). Similarly, the 2-year estimate for MOD-PFS was 90.3% (95% CI, 80.4–100%) for rapid hematologic dFLC response patients and 55.6% (95% CI, 29.3–100%) for slow hematologic dFLC response patients (*P* = 0.07).

## Discussion

Our study demonstrates that treatment with daratumumab, bortezomib, and dexamethasone leads to a rapid decrease in dFLC levels by D7 post-treatment. This reduction can predict the achievement of first-line complete hematologic response. We introduce the novel concept of rapid hematologic dFLC response, which is of clinical significance. In our cohort, rapid hematologic dFLC response is defined as a reduction in dFLC levels exceeding 67% in C1D7.

A rapid hematologic dFLC response in C1D7 suggest a high possibility of achieving CHR following treatment with daratumumab, bortezomib, and dexamethasone. Compared to achieving a VGRP or PR, achieving CHR can potentially lead to an improved organ response and survival benefit [19]. Considering that the median time to achieve CHR with Dara-BCD is 85 days (range, 29–179 days) [10], the primary endpoint set as CHR within 6 months is reasonable for this study.

Our results are consistent with the dynamics of daratumumab-based treatment in AL amyloidosis. Dara-BCD treatment for systemic light-chain amyloidosis achieved a CHR rate of nearly 60%, with a median onset time of 9 days [3]. A pivotal phase 2 study conducted at multiple centres in France and Italy evaluated the efficacy of daratumumab monotherapy for relapsed/refractory AL amyloidosis [9]. The study found that the median time to the onset of the hematologic response was 7 days. Among the 40 patients studied, the average decrease in dFLC after a single dose of daratumumab was 49%. Within the responder group, the median decrease in dFLC after one dose of daratumumab was 63%. 19 out of 40 patients achieved ≥ PR efficacy after one dose of daratumumab. Of note, all patients who did not achieve PR efficacy after one cycle of treatment did not experience further improvement in hematologic response with subsequent treatments. In this study, we quantified the dFLC decrease in C1D7 using ROC curve analysis. Our analysis revealed that a decrease exceeding 67% demonstrates both high sensitivity and specificity for achieving first line CHR.

In this retrospective study, 48 patients initially received DaraBD treatment with or without cyclophosphamide, while seven patients received daratumumab and dexamethasone on C1D1, followed by bortezomib in subsequent cycles. All seven of these cases were in advanced cardiac stages. Among these seven patients, four did not achieve CHR within 6 months, and the magnitude of decrease after one shot of daratumumab ranged from 15.2 to 58.3%. Three patients achieved CHR, with all of them experiencing a decrease of more than 67% after just one dose of daratumumab. It is clinically significant to re-explore dFLC changes in C1D7 for advanced cardiac stages with the initial use of daratumumab and subsequent addition of bortezomib. This treatment strategy is particularly important in patients when bortezomib use is limited, such as those in advanced cardiac stages [17].

In this study cohort, 32 patients did not undergo treatment with cyclophosphamide. Previous research in AL amyloidosis showed that addition of cyclophosphamide to bortezomib and dexamethasone did not significantly improve long-term overall survival or time to next treatment [23]. We believe that potential bias resulting from the exclusion of cyclophosphamide is minimal.

Our treatment protocol, especially the adjustment of the treatment regimen, differs from that of the ANDROMEDA trial. In the ANDROMEDA clinical trial, the Daratumumab-BCD regimen was continued unless there was hematological progression or the patient experienced end-stage cardiac or renal failure. In our clinical practice, we changed the treatment regimen when the patient did not achieve at least a PR after one cycle, or a VGPR after four cycles, except in cases where there was no suitable alternative regimen available for specific patient or if the patient had already achieved the desired organ response. This approach is consistent with the mSMART consensus from the Mayo Clinic [15].

Several limitations of our study arise from its retrospective nature and the small sample size. Additionally, the dosage of bortezomib, from 0.7 mg/m^2^ to 1.3mg/m^2^, is not standardized. Therefore, our conclusions should be validated in large-scale clinical studies, such as the ADNROMEDA trial.

In summary, we propose a new concept, “rapid hematologic dFLC response,” in AL amyloidosis, based on the treatment with daratumumab, bortezomib, and dexamethasone. Rapid hematologic dFLC response can predict the likelihood of achieving complete hematological response within 6 months. With the increasing availability of treatment options [24–27], precise and dynamically adjusted treatment based on rapid hematologic dFLC response can lead to higher rates of hematologic complete response, organ response, and overall survival.

## Electronic Supplementary Material

Below is the link to the electronic supplementary material.

Supplementary Material 1

## Data Availability

Data are available upon reasonable request.
