# Supplementary material for: Early dFLC response by C1D7 predicts complete hematologic response in systemic AL amyloidosis
Source: Ann Hematol. 2024 Nov 12;104(1):617–25. doi: 10.1007/s00277-024-06077-0 (PMC11868242; doi:10.1007/s00277-024-06077-0)
Supplement: Supplementary file 1 — Supplementary Material 1 [file 277_2024_6077_MOESM1_ESM.docx]

Supplementary Table 1. Grading of organ response in Rapid Respond group and Slow Respond group

|  | Rapid Responders (n/%) | Slow Responders (n/%) | P |
| --- | --- | --- | --- |
| Cardiac | 25 | 16 |  |
| NR | 7(28%) | 9(56.2%) | 0.070 |
| PR | 5(20%) | 1(6.25%) | 0.376 |
| VGPR | 10(40%) | 5(31.3%) | 0.570 |
| CR | 3(12%) | 1(6.25%) | 1.000 |
| Renal | 25 | 11 |  |
| NR | 7(28%) | 8(72.7%) | 0.012 |
| PR | 3(12%) | 1(9.1%) | 1.000 |
| VGPR | 10(40%) | 1(9.1%) | 0.116 |
| CR | 5(20%) | 1(9.1%) | 0.643 |

NR, no response; PR, partial response; VGPR, very good partial response; CR, complete response.
